# Low Fatization of High-Fat Surimi-Based Products: Optimization of the Application of Protein Matrix Fat Substitution Methods

**DOI:** 10.3390/gels9090724

**Published:** 2023-09-06

**Authors:** Guangyu Yan, Lei Yu, Xiaoting Chen, Zhiyu Liu, Hui Chen

**Affiliations:** 1 Xiamen Ocean Vocational College, Xiamen 361102, China; guangyu0502@163.com (G.Y.); coloso@sina.com (L.Y.); 2 Fisheries Research Institute of Fujian, Xiamen 361013, China; xtchen@jmu.edu.cn (X.C.); negroliu@163.com (Z.L.); 3 Technology Innovation Center for Exploitation of Marine Biological Resources, Third Institute of Oceanography, Ministry of Natural Resources, Xiamen 361005, China

**Keywords:** low-fat *Nemipterus virgatus* fish sausage, low fatization of high-fat foods, electronic nose, sensory evaluation, gel strength

## Abstract

The low fatization of high-fat foods is a significant trend that will impact the future developments of food products. Consumers have regarded health attributes as a critical indicator for purchasing food. In this study, enzyme-modified soy protein isolate, sea fish collagen, and ovalbumin were used to prepare the composite fat substitute for the protein matrix. This matrix was applied to the traditional surimi-based product *Nemipterus virgatus* fish sausage to replace the exogenous fat, and a new type of low-fat fish sausage was developed. This change is expected to reduce the exogenous fat in the traditional fish sausage without reducing the flavor and sensory quality of the original product. The results showed that taking the sensory evaluation and gel strength value of the product as indicators, the optimal ratio of compound fat substitute (enzyme-modified soy protein isolate:sea fish collagen:ovalbumin) was 2:1:3 when using the orthogonal test method for the first time. In the next step, with compound fat substitutes, exogenous fats and transglutaminase as the main factors, single factor and response surface method were used to explore the best formula of new low-fat *Nemipterus virgatus* fish sausage. The results showed that the best gel strength and sensory evaluation scores were obtained when the compound fat substitute, TGase, and exogenous fat were 0.59 g, 0.245 g, and 8.03 g, respectively. The optimal formulation of the low-fat *Nemipterus virgatus* fish sausage was obtained as follows: surimi, 67.52%; complex fat substitute, 0.66%; TGase, 0.28%; fat, 9.04%; starch, 6.75%; sugar, 3.94%; salt, 2.25%; monosodium glutamate, 0.23%; I&G, 0.34%; and water, 9%. Compared with the traditional fish sausage, the content of exogenous fat in the new, low-fat *Nemipterus virgatus* fish sausage was reduced by 54.8%. Meanwhile, the sensory score of fish sausage was increased by 21.79%, maintaining its good flavor and sensory quality. This study provides an important reference value for developing new low-fat surimi-based products.

## 1. Introduction

The low fatization of high-fat foods is a significant trend that will impact the future developments of food products. High-fat and high-calorie diets are closely related to the health risks caused by obesity. For example, chronic diseases such as cardiovascular disease and cancer are positively related to the amount of fat consumed in the human body [1,2,3]. Therefore, the development of low-fat foods has become an important method to reduce human disease. Low-fat foods, especially low-fat meat products, have been valued by many countries. For example, Canada has incorporated low-fat diets into the law [4], and the United States has developed over 2000 fat substitutes to develop low-fat meat products. According to experts’ estimates, the world’s annual sales of low-fat meat products will increase by 25.5%. From 2017 to 2021, the number of registered low-fat healthy light food enterprises in China has significantly increased, with a cumulative increase of 64% of the total number of new enterprises. However, some consumers are unwilling to reduce sensory quality to improve nutritional value [5]. Therefore, on the basis of the obvious reduction of fat in food while ensuring the taste, texture and other quality characteristics of food, continuous research on new fat substitutes and the development of functional, nutritional, comprehensive, and healthy low-fat foods have profound developmental significance and broad market prospects.

In recent years, the global production and consumption of surimi-based products have shown a rapid growth trend, and they are also among the fastest-growing aquatic processed products in China. In 2021, the processing output of the national surimi-based products industry was 1.334 million tons, with Fujian Province accounting for about one-third of the production, ranking first in the country. The apparent consumption of surimi-based products in China was 1.2219 million tons. In 2021, the market size of surimi-based products in China reached CNY 18.732 billion, an increase of 11.99% compared to CNY 16.727 billion in 2020. With the formation of a large-scale industrial competition pattern in the surimi-based product industry, continuously improving product quality and developing new high-quality surimi-based products are the best countermeasures for our province to address the challenges of the surimi-based product industry. Compared to other meat products, such as livestock and poultry, surimi is very healthy because it contains multiple vitamins, rich protein, and has low fat content. Due to the relatively low fat content of fish meat, to ensure the taste and texture characteristics of the product [6] in actual production, improve the flavor and sensory quality of the product, and reduce production costs, a certain proportion of exogenous fat is often added in the production process of surimi-based products. Market research results show that many surimi-based products sold on the market currently have a fat ratio of 20% to 30%, or even higher. Data from the US Dietary Guidelines and the Outline of Food and Nutrition Development in China (2014–2020) show that fat provides less than 30% of total calories in People’s Daily diets [7]. High fat and cholesterol in traditional surimi-based products limit their consumption in some people. Research has shown that enhancing the health attributes of meat products and reducing fat content positively impacts consumers’ choice of meat products [8]. Therefore, new low-fat surimi-based products are emerging.

Fat substitutes have low fat characteristics, perfectly fitting current dietary and health concepts. Protein matrix “fat substitutes” are natural proteins that, after denaturation treatment (pH, salt, metal, etc.), expose hydrophobic groups on the molecular surface, exhibiting hydrophobic properties similar to those of oils. At the same time, the micronized protein undergoes hydration, forming protein particles with a diameter of less than 10 microns, effectively simulating the smooth and delicate taste of fat. Protein-based fat substitutes have a positive impact on low-fat foods, with the following advantages in terms of flavor interactions and fat substitution: improving quality, nutritional value, and functional characteristics and reducing cholesterol, fat content, and energy density of low-fat foods. Common proteins include soybean protein, milk protein, casein, ovalbumin, collagen, etc.

The soybean protein isolate (SPI) has a high nutritional value and a low price, making it an ideal substitute for animal protein. Due to its rich protein content and important role in food structure, soy protein isolate has a structure similar to fat characteristics, and has become one of the food ingredients and commonly used fat substitute substrates, which is widely used in protein-based food formulations. The modified soy protein isolate and water form a dispersed liquid, which can form a gel under certain conditions. It has physical and chemical properties similar to fat and is increasingly used in processing of low-fat meat products. It was found that enzyme-modified soy protein could effectively reduce the caloric and cholesterol intake of meat products and improve the water-holding capacity and hardness of meat products [9,10]. Some studies have made use of enzymatically modified soy protein isolate to produce meat products with better flavor and taste, less fat, less calories and no beany flavor. Soy protein with good emulsification and dispersion can improve the thermal stability of the product, minimize cooking losses, and improve product yield, flavor, and appearance characteristics [11,12]. In the process of making Asian traditional rice meat dumplings, Sun Peiran added enzymolysis soybean flavor substances to improve the viscoelasticity of rice meat dumplings [13]. MeMinds [14] and others made beef mince pies with good flavor and taste by separating proteolysis hydrolysates from soybeans. The soybean-separated proteolysis hydrolysate can reduce the fat in meat products by 5–75% and the heat by 20–70% while ensuring the due sensory quality of the products. This not only avoids an unpleasant beany smell but also has an antioxidant effect that can slow the deterioration of the color and flavor of the products in the process of frozen storage. Ahmad [15] added 0%, 15%, and 25% soy protein isolates to low-fat beef emulsion sausage, which significantly improved the water content, color, and sensory quality of low-fat sausage under the same storage time compared to the control.

The unique quality of collagen makes it a functional substance and nutritional component in many foods. In terms of health functions, it has the effects of increasing bone density, improving bone defects, lowering blood pressure, and has good biocompatibility and antioxidant activity. This is especially notable for marine collagen. Because of its unique amino acid composition, it has incomparable advantages over terrestrial collagen in terms of gel properties, safety, etc. Wu [16] found that collagen can significantly enhance the folding grade of surimi and minced meat products, forming a more solid three-dimensional reticulated protein gel structure, thus enhancing their gel properties. Meanwhile, collagen peptides of certain molecular weights had a strong inhibitory effect on ice crystal recrystallization and reduced the freezing pressure effect on water flow properties, thus protecting surimi-based products from a loss of fixed and bound water during freezing and effectively maintaining their structural stability [17]. In addition, surimi-based products containing collagen peptides had better properties, including shear thinning behavior, better recovery, and better mechanical properties [18]. Dong [19] added acid-soluble collagen and enzyme-soluble collagen extracted from catfish skin to grass carp minced fish and significantly improved the gel strength of minced fish. Weng [20] showed that collagen peptides containing α-peptide chains derived from tilapia fish skin could improve the gel strength, texture, and water retention properties of surimi-based products and had no significant effect on the color of surimi-based products. Different concentrations of fish scale gum were added to surimi, which resulted in a significant decrease in water loss and a considerable increase in the whiteness of surimi gels [21]. Cod collagen can be used as a meat product improver to improve the softness and water-holding power of meat products [22].

Egg white powder is widely used in the food industry because of its many positive functional properties, such as foaming, emulsification, and gelation, flavoring effects and water retention [23]. Its gelation property is mostly used in the production of surimi-based products and meat products [24]. The protein in egg whites is mainly ovalbumin [25]. Ovalbumin is a spherical monomeric phosphoglycoprotein with a strong gelation ability [26]. During gelation, ovalbumin unfolds in a molten spherical structure and then aggregates to form a three-dimensional gel network structure [27]. Ovalbumin is a non-muscle protein. It contains hydrophilic groups that will absorb water and swell during the collapse of surimi-based products to improve the gel strength; non-muscle protein can directly interact with myogenic fibrin to fill the gel network and enhance the texture of surimi [28]. By comparing the effects of starch, protein, and carrageenan on the gel properties of surimi, Chen [29] found that egg whites were the most effective in improving the gel properties of surimi. Many studies found that egg white protein significantly improved the gel properties of surimi, thus improving the quality of surimi-based products [30,31,32,33,34].

Fujian Province is extremely rich in marine fishery resources, and a large amount of leftover materials, such as skin, bones, scales, fins, etc., generated during the processing of aquatic products contain rich collagen. Due to the differences in amino acid composition and cross-linking degree, marine collagen has better processing characteristics than terrestrial collagen, such as better gel properties, dispersibility, water absorption, water retention, emulsification, and other processing characteristics, and has higher safety characteristics, such as low antigenicity and low allergens.

This study considers the rich marine collagen, soy protein isolate peptide, and ovalbumin in Fujian Province as raw materials and the gel strength and sensory score as indicators. Then, it obtains the best compound fat substitutes via orthogonal analysis. Further, compound fat substitutes were added to surimi-based products in different proportions to replace the exogenous fat in the initial formula. The quality analysis of new low-fat surimi-based products supplemented with flavor analysis (electronic nose) considers the gel strength and sensory score as indicators. Based on a single-factor experiment, the optimal formula of low-fat surimi-based products is obtained via response surface analysis. The purpose of this study is to develop a new low-fat surimi-based product by reducing the content of exogenous fat (reduce exogenous fat content by 20%) while maintaining its good flavor and sensory quality. This product not only meets consumers’ requirements for low-fat and delicious food but also brings more health benefits to consumers, meeting the needs of modern people for a healthy diet with significant social and economic benefits.

The implementation of this research helps to expand the application fields of fish processing by-products and has a positive promoting effect on the healthy development of the aquatic processing industry and the surimi-based product industry in our province. Implementing the findings from this study can promote the development of low-fat and healthy products, achieve the high-value development and utilization of low-value marine biological resources, and innovate the types and quality of surimi-based products, greatly promoting the transformation of regional resource advantages into economic advantages.

## 2. Materials and Methods

### 2.1. Materials and Equipment

#### 2.1.1. Experimental Materials

*Nemipterus virgatus* surimi was provided by Fu Jian Anjoy Foods Co., Ltd. (Xiamen, China); soybean isolate protein was supplied by Wanlida Biotechnology Co., Ltd. (Zhangzhou, China); I&G (IMP + GMP, Disodium Inosine-5′-Monophosphate 50% + Disodium guanosine-5′-Monophosphate 50%) was obtained from Hijer (Liaocheng) Biotechnology Co., Ltd. (Liaocheng, China); Monosodium glutamate (MSG), salt, sugar, fat, and potato starch were purchased from the Xia Shang Supermarket. Fish collagen was homemade in the laboratory. TGase was obtained from Nanning Donghenghuadao Biotechnology Co., Ltd. (Nanning, China). Ovalbumin was obtained from Guangdong Yitianyuan Biotechnology Co., Ltd. (Guangzhou, China). Plastic casing (food grade material, PA/PE material) was purchased from supermarkets.

#### 2.1.2. Experimental Equipment

TX-PLUS mass spectrometer (Stable Micro System, Godalming, UK), H1650-W high-speed centrifuge (Hunan Xiangyi Laboratory Instrument Development Co., Ltd., HuaiHua, China), PEN3 electronic nose (Airsense, Schwerin, Germany); HH-6 digital display thermostatic water bath (Guohua Electric Co., Ltd., Hongkong, China), ADCI automatic colorimeter (Beijing Tentec Instruments Ltd., Beijing, China), and YC-3L enema machine (Shanghai Yechang Food Machinery Co., Ltd., Shanghai, China).

### 2.2. Experimental Method

#### 2.2.1. Initial Formulation of Low-Fat *Nemipterus virgatus* Fish Sausage

Traditional fish sausage original formula: The amounts of fish surimi is 60 g, fat is 20 g, starch is 6 g, sugar is 3.5 g, salt is 2 g, MSG is 0.2 g, I&G is 0.3 g, and water is 8 g. The proportion of each component in the fish sausage is fish surimi, 60%; fat, 20%; starch, 6%; sugar, 3.5%; salt, 2%; monosodium glutamate, 0.20%; I&G, 0.30%; and water, 8%.

#### 2.2.2. Processing Process and Operation Points of Low-Fat *Nemipterus virgatus* Fish Sausage

(1) Process flow: Pre-treatment of surimi mixture → chopping → placing the surimi into the casing → heating and cooling → cutting.

(2) Process operation points.

The surimi and the shredded exogenous fat (lard) were put into the chopper and chopped for 4 min, then the compound fat substitutes (fish collagen peptide, soy protein peptide, ovalbumin) were added and chopped for 1 min, then starch, sugar, salt, MSG, I&G, and water were added and chopped for 4 min for seasoning. Finally, TGase was added and mixed manually for about 1 min until it became uniform.

To strictly control the temperature during the entire chopping process, all auxiliary materials should be placed in the cooling room for at least 24 h so that the temperature falls to below 10 °C. The temperature during the chopping operation should not be higher than 10 °C, and the operating environment temperature should be controlled so that it is below 15 °C. After chopping, the seasoned surimi mixture can be packed and stored at 4 °C.

After placing the surimi mixture into the casing, the fish sausage formed in the casing was put into a constant temperature water bath at 40 °C for 30 min to shape, then put into a constant temperature water bath at 90 °C for 20 min to mature, and then immediately placed into ice water for 30 min to fully cool it. Finally, it was placed in a refrigerator at 4 °C for 12~24 h to rest and equilibrate before use.

### 2.3. Experimental Design of Low-Fat Nemipterus virgatus Fish Sausage Formulation

#### 2.3.1. Optimization of the Composition of Compound Fat Substitutes

The suitable addition range of collagen, soy protein peptide, and ovalbumin in the complex fat substitute was obtained based on the previous single-factor experiment. To further optimize the composition of collagen, soy protein peptide, and ovalbumin in the complex fat substitute (based on a 20% reduction in the addition of exogenous fat compared with the original formulation), a 3-factor, 3-level orthogonal test (L_9_ (4^3^)) was designed to determine the optimal amount of collagen, soy protein peptide, and ovalbumin to be added to the composite fat substitute, using the gel strength and sensory quality of low-fat fish sausage as evaluation indexes.

#### 2.3.2. Experimental Study of Low-Fat *Nemipterus virgatus* Fish Sausage Formulation

(1) Single-factor experimental design of low-fat *Nemipterus virgatus* fish sausage formulation.

The effects of the addition of compound fat substitute, TGase, and exogenous fat on the gel strength and sensory quality of fish sausage were investigated using single-factor tests under constant process conditions and other parameters to provide a basis to optimize the low-fat fish sausage process formulation.

(2) Response surface experimental design of low-fat *Nemipterus virgatus* fish sausage formulation.

Based on the single-factor test, to investigate the effects of compound fat substitution addition, TGase enzyme addition, and exogenous fat addition on the gel strength and sensory quality of fish sausage, a 3-factor, 3-level response surface test was designed to determine the optimal formulation of low-fat fish sausage process, using the gel strength and sensory quality of fish sausage as indicators.

### 2.4. Determination of Gel Strength [35]

Gel strength is one of the basic and important indexes to measure the quality of surimi-based products. The cooled fish sausage stored at 4 °C for 24 h was taken out of the refrigerator, the plastic casing was peeled off, and the sausage was cut into 25 mm × 20 mm (diameter × height) cylindrical sections, which required a neat and smooth cut surface. The gel strength of the fish sausage was measured via TX-PLUS mass spectrometer at room temperature. The spherical probe P/0.5 S of the mass spectrometer was used for the rupture experiment, and the test speed was set to 1 mm/s, 50% of the sample thickness was punctured, and the trigger force was 5 g. The first peak of the obtained puncture curve was the rupture strength, and the rupture distance corresponding to the rupture strength was the rupture depth. At least 6 parallels were made for each sample, and the average value was taken. The gel strength (g·cm) = breaking strength (g) × breaking depth (cm).

### 2.5. Sensory Scoring Criteria for Low-Fat Nemipterus virgatus Fish Sausage

Sensory evaluation was used to represent the overall quality of surimi-based products. The scoring method was employed to comprehensively evaluate the quality of *Nemipterus virgatus* fish sausage [36,37]. The sample was cut into 2 cm segments. Seven food professionals who had received sensory training were invited to form an evaluation team. Sensory evaluation was conducted in the sensory laboratory according to the evaluation criteria in Table 1. The evaluation indicators are taste, odor, color, tissue morphology, and elasticity. Each item is scored at 20 points; the higher the score, the better the effect. The average score of the seven assessors was used as the rating for each indicator. During the evaluation stage, the assessor was allowed to approach or consume standard reference materials and use purified water to remove residual flavors in the mouth between evaluations.

### 2.6. Flavor Analysis—Electronic Nose Analysis Method

As a device for detecting food flavor, an electronic nose can obtain results consistent with human sensory evaluation and has been widely used in food quality identification, flavor analysis, component detection, brand classification, freshness detection, product process improvement, and other fields [38,39,40]. Compared with manual detection, it has the advantages of convenience, objectivity, and good stability.

First, 20 g of *Nemipterus virgatus* fish sausages were weighed then placed in a headspace bottle. We let them stand at room temperature (25 ± 1 °C) for 30–40 min until the volatile matter in the top space reached equilibrium. An electronic nose sampling needle inserts the matter into the headspace bottle through the cling film for headspace sampling and detection. The detection method adopts the headspace aspiration method. The process is repeated six times for each sample. The measurement parameters are as follows: sensor cleaning time of 80 s, reset time of 10 s, sample preparation time of 5 s, sample testing time of 100 s, cleaning time of 200 s, and internal flow rate of 150 mL/min.

### 2.7. Statistical Analysis

SPSS 17.0 was used for the analysis of variance, one-way ANOVA was used for difference analysis, and Duncan multiple comparisons were used for significance analysis. In this study, the *p*-value was used to analyze the significance level of the difference in mean values. Indicators with *p* < 0.05 are statistically different. *p* < 0.01 indicates a significant statistical difference, and *p* < 0.001 indicates an extremely significant statistical difference.

Electronic nose data are collected and processed using Winmuster software (Version 1.6.2), and a cluster discriminant analysis is performed on different samples using principal component analysis (PCA). PCA is a method of applying variance decomposition to reduce the dimensionality of the extracted multidimensional data information, extract the most important elements and structures in the data, and linearly classify the reduced feature vectors. Finally, a two-dimensional scatter plot is displayed on the scatter plot and analyzed via PCA. Principal component 1 (PC1) and principal component 2 (PC2) contain the contribution rates of the first and second principal components obtained in PCA conversion. The larger the contribution rate, the better the main component can reflect the original information of multiple indicators [41].

## 3. Results and Discussion

### 3.1. Test Results and Analysis of Low-Fat Nemipterus virgatus Fish Sausage Formulation

#### 3.1.1. Determination of the Composition of the Compound Fat Substitution

Based on a 20% (4 g) reduction in the exogenous fat in traditional fish sausages, the optimal composition ratio of fish collagen, soy protein peptide, and ovalbumin in composite fat substitutes was explored. Preliminary experiment: First, through a single-factor experiment, the optimal dosage of fish collagen, soy protein peptide, and ovalbumin was determined to be 0.20 g, 0.10 g, and 0.20 g, respectively.

To further optimize the composition of fish collagen, soy protein peptide, and ovalbumin in the compound fat substitute, based on the suitable addition amounts of 0.20 g, 0.10 g, and 0.20 g obtained from the previous single-factor test, the orthogonal test was conducted to determine the optimal amounts of each in the compound fat substitute as the test factors. The optimum amounts of collagen, soy protein peptide, and ovalbumin in the compound fat substitute were determined based on the gel strength and sensory quality.

The sensory scores and gel strength are two significant evaluation indicators in evaluating low-fat fish sausage quality. Therefore, a double index was used for the extreme difference analysis, which was assigned a weight of 80% for the sensory score and 20% for the gel strength, and the overall evaluation score for the quality of the fish sausage was calculated.

The specific calculation is as follows: first, the value of each indicator for each of the nine samples is divided by the largest value within the nine samples for that chemical component. It is then multiplied by the weighting factor to obtain the contribution value of that indicator in the composite score of that sample. The contribution value of each indicator in the same sample was summed to give the composite score of that sample [42,43]. For example, the composite score of sample 2 = (170.186/555.559) × 0.20 + (63.15/78.08) × 0.80 = 0.7024, and the results are shown in Table 2. The orthogonal design test factors and levels are shown in Table 2, the test design and results are shown in Table 3, and the analysis of variance is shown in Table 4.

As shown in the results of the orthogonal experiments in Table 3, the main order of the influence of the factors on the three additions of fish collagen, soy protein peptide, and ovalbumin in the compound fat substitute is A > C > B, i.e., fish collagen > ovalbumin > soy protein peptide. The results of the ANOVA in Table 4 show that the effects of the three factors on the combined scores of the ratios in the composite fat substitutes were not significant, and the best level was taken by considering the main factors in an integrated manner. The other factors were obtained as A_2_B_2_C_3_ according to the consideration of cost saving and profitability of each factor.

#### 3.1.2. Validation Test

The experiments were repeated three times according to the results of orthogonal experiments on the A_2_B_2_C_3_ formula to verify the rationality and stability of the additions of fish collagen, soy protein peptide, and egg white protein in the compound fat substitution. A (fat substitute), B (TGase), and C (fat) were set to 0.2 g, 0.1 g and 0.3 g, respectively, under these conditions. The gel strength and sensory evaluation of low-fat fish sausage was measured and scored. The average value was obtained from three repetitions of the experiment. The average gel strength was 563.20 g·cm and the average sensory score was 77.27. The overall score was calculated as 0.9706, which was 1.52% different from the predicted value of the model, and the prediction condition was reliable.

The analysis of the orthogonal experimental results showed that the optimal amounts of fish collagen, soy protein peptide, and ovalbumin in the complex fat substitution in low-fat *Nemipterus virgatus* fish sausage were 0.2 g, 0.1 g, and 0.3 g, respectively, i.e., the ratios of fish collagen, soy protein peptide, and ovalbumin in the complex fat substitution were 2:1:3.

### 3.2. Determination of Single-Factor Parameters for the Process Formulation of Low-Fat Nemipterus virgatus Fish Sausage

#### 3.2.1. Effect of Compound Fat Substitution Addition on Gel Strength and Sensory Quality of Low-Fat *Nemipterus virgatus* Fish Sausage

**Figure 1:**
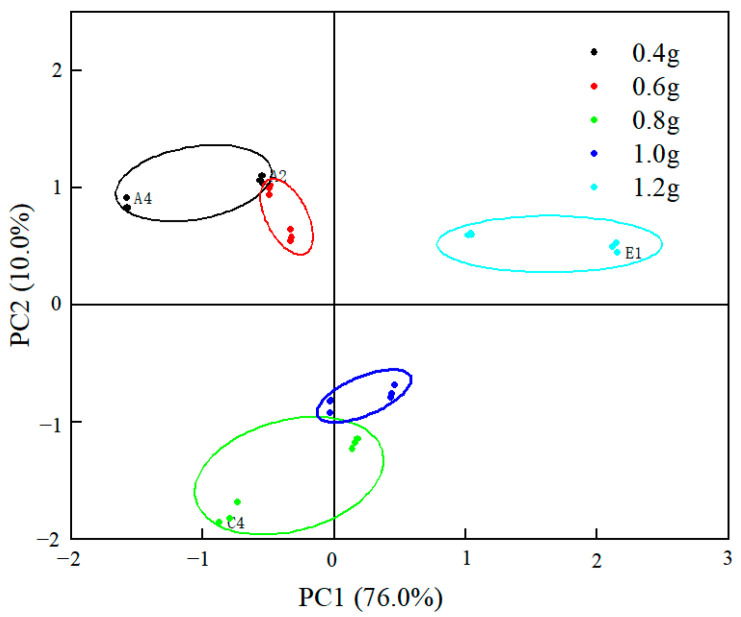
PCA plot of PC1 against PC2 for low-fat fish sausage with different fat substitute addition.

Based on a 20% reduction in the addition of exogenous fat in traditional fish sausage formulas, the compound fat substitutes were added in the amounts of 0 g (as a control group), 0.4 g, 0.6 g, 0.8 g, 1.0 g, and 1.2 g. The test results are shown in Table 5 and Figure 1.

Because soy protein isolate, collagen, and ovalbumin have good emulsifying, gelling, and water-holding properties, adding a certain amount of soy protein isolate, collagen, and ovalbumin during surimi-based product processing can effectively improve their gel properties and sensory quality. When the amount of collagen in *Tilapia* sausages is 15% (*w*/*v* is 6.67%), the tilapia sausages are complete in shape, elastic, dense in cross-section holes, and good in taste and flavor [44]. Moreover, 6% soybean protein can improve the gel strength of Grass Carp sausage [32]. When the addition of ovalbumin was 7% and soybean protein was 2%, the elasticity of surimi-based products could be significantly improved, and the quality of surimi-based products could be effectively improved [45]. The results of this study also showed that the gel strength of low-fat fish sausage increased with the increase in the amount of compound fat substitute (Table 5).

However, when the amount of compound fat substitute was more than 0.8 g, the increase of gel strength became less noticeable. The research results of Zhou [30] and Xu [33] also proved this conclusion. The results showed that the appropriate amounts of egg white protein powder and soybean protein powder could improve the quality of fish balls, but the gel strength of fish balls did not increase significantly with increases in the added amount of egg white protein powder and soybean protein powder. The reason should be that, compared with the fish sausage without soy protein isolate, the degradation effect of fish sausages with soy protein isolate on the muscle ball heavy chain at the later stage of gel formation is not obvious [12,46].

Table 5 shows that the sensory score of the low-fat fish sausage increased first and then decreased with the increasing amount of fat substitutes. The change trend of the comprehensive score was consistent with the sensory score and reached the maximum when the amount of compound fat substitute was 0.6 g, which was significantly higher than that of other groups (*p* < 0.05). This may be because the protein flavor of excessive soy protein isolate suppresses the flavor of surimi, reduces its flavor, and causes a loose product structure [47].

The principal component effects of the e-nose differentiation of fish sausage samples with different compound fat substitution additions are shown in Figure 1, and the variance contributions of the first and second principal components are 76.0% and 10.0%, respectively, accumulating to 86.0%. This result indicates that PC1 and PC2 already contain most of the information and can reflect the overall information of the samples. The first principal component mainly reflects the differences between the samples. As shown in Figure 1, the low-fat fish sausage samples with the addition of 0.4 g and 0.6 g of compound fat substitute were located in the upper left side of PC1, and both of them were clearly separated from the other three groups of samples, indicating that the flavor of both of them was outstanding, and the appropriate additional amount of fat substitute was determined to be 0.6 g by combining with the gel strength and sensory evaluation results.

#### 3.2.2. Effect of Exogenous Fat Addition on Gel Strength and Sensory Quality of *Nemipterus virgatus* Fish Sausage

**Figure 2:**
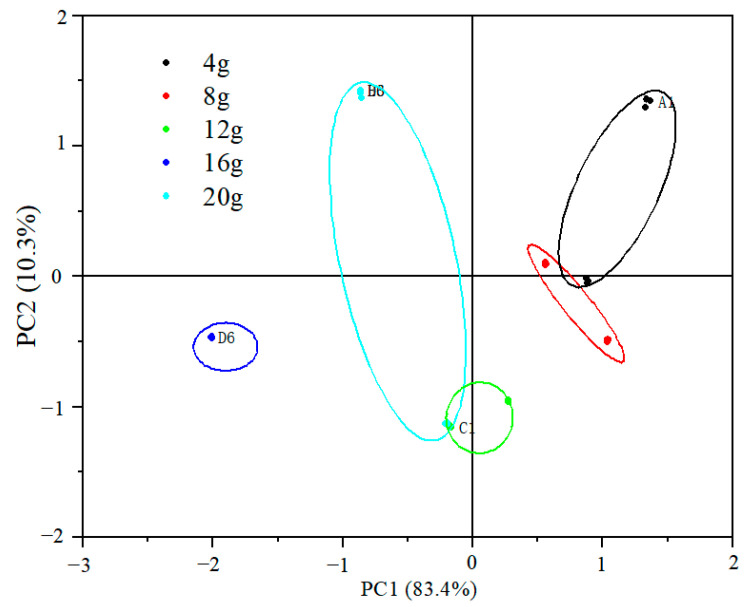
PCA plot of PC1 against PC2 for low-fat fish sausage with different fat addition.

The exogenous fat was added in the amounts of 20.0 g (as a control group), 16.0 g, 12.0 g, 8.0 g, and 4.0 g, and the test results are shown in Table 6 and Figure 2.

As shown in Table 6, the highest sensory score of the fish sausage was obtained when the amount of fat added was 8.0 g. The comprehensive score was also significantly higher than the other groups (*p* < 0.05). With the increase in the amount of exogenous fat, the strength value of the gel still increased, but the increase was not obvious, and the sense organ began to become slightly rough. A similar conclusion was also found when studying the effect of pork fat on the quality of surimi-based products by Wang [48]. When 6% fat is added to surimi-based products, the elasticity of surimi-based products can be improved, the freezing water loss rate of surimi-based products can be reduced, the whiteness of the products can be significantly improved, and the flavor and taste of the products can also be improved. However, if the amount of fat is too high, the oil taste of the products will be too heavy, and the taste will become worse.

An appropriate addition of exogenous fat can simultaneously give fish sausage the freshness of fish and the fragrance of pork. It can also wrap many flavorful substances in a fish sausage in its oil phase so that it is not easy to disperse, increasing its oily and flavorful taste, and can play a certain role in suppressing the fishy taste in surimi and better maintain the elasticity of surimi-based products [19,20], prevent hardening, and prolong their shelf lives. However, if too much fat is added, the products will have too much of a fatty taste and will cause the internal structure of the fish sausage to disperse, producing pores or even holes. Thus, the free fat will precipitate, and the taste will deteriorate. 

The variance contributions of the two principal components of the low-fat fish sausage samples with different fat additions in Figure 2 were 83.4% and 10.3%, respectively, reaching 93.6% cumulatively. When the fat addition amounts of 12.0 g, 16.0 g, and 20 g of low-fat *Nemipterus virgatus* fish sausage samples were located in separate areas and were widely spaced from the other three groups, it was indicated that the flavor of these two groups was relatively prominent compared with the other three groups. Combined with the gel strength and sensory evaluation results, the appropriate amount of fat addition was determined to be 8.0 g. The specific amount of the fat added was determined via response surface testing.

#### 3.2.3. Effect of TGase Addition on Gel Strength and Sensory Quality of Low-Fat *Nemipterus virgatus* Fish Sausage

**Figure 3:**
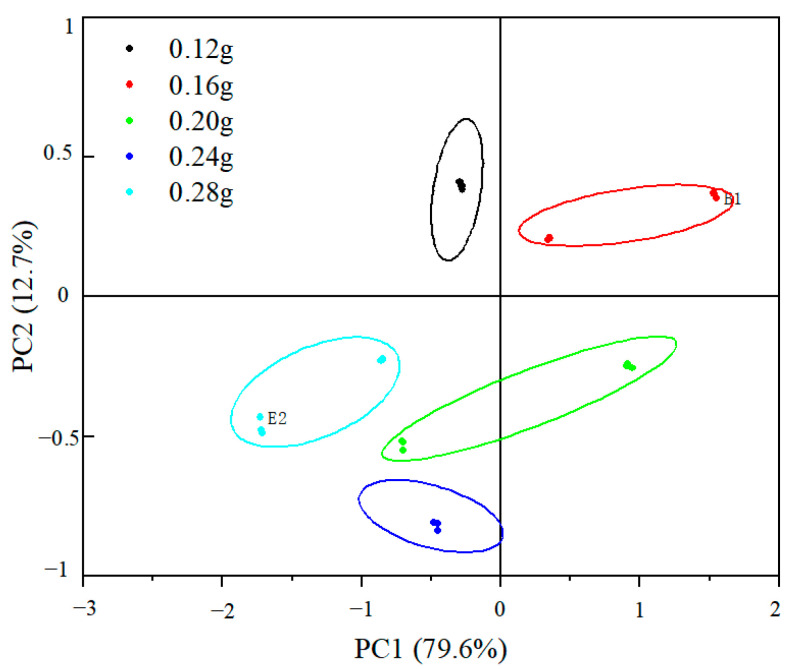
PCA plot of PC1 against PC2 for low-fat fish sausage with different TGase addition.

Based on a 20% reduction in the addition of exogenous fat in traditional fish sausage formulas. TGase was added in the amounts of 0 g (as a control group), 0.12 g, 0.16 g, 0.20 g, 0.24 g, and 0.28 g. The test results are shown in Table 7 and Figure 3.

Although fish muscle also contains endogenous TGase, which plays a certain role in the gelation process of surimi, the content is small, and it often needs to be added externally to improve the gel strength of surimi. Transglutaminase (TGase) is a transferase that catalyzes the acyl transfer reaction. It can catalyze the transformation of lysine on protein ε-Amino glutamic acid γ-Hydroxyamide groups form covalent bonds to promote covalent cross-linking between or within protein molecules, thereby enhancing the gel strength of products and improving the quality of products [49].

As shown in Table 7, with the increase in TGase addition, the tissue structure of fish sausage became firmer and more elastic. When the addition of TGase was 0.24 g, the gel strength and sensory score of the fish sausage were the highest, and the comprehensive score reached the maximum, significantly higher than other groups (*p* < 0.05). After that, the gel characteristics and sensory score of fish sausage decreased with the increase in TGase addition. The results of this study are consistent with those of Jiadan et al. [50]. These research results showed that TGase could reduce the energy barrier required for surimi gel formation and make ε-(γ-glutamyl) lysine covalent bonds easier to form. With the increase in TGase content, the breaking force and water-holding capacity of surimi gel first increased and then decreased. However, some studies suggest that the effect of TGase on the gel properties of surimi varies with the fish species. For example, when the addition of TGase was 0.2 U·g^−1^, the gel properties of Spanish mackerel surimi were the highest, and the gel properties of the surimi decreased with the increase in TGase. However, for mackerel and sardines, the gel properties of surimi increased with the increase in TGase content [51].

Figure 3 shows that the variance contribution of the first and second principal components of the samples with different TGase addition levels of low-fat fish sausage accumulated to 92.3% (79.6% and 12.7%, respectively), and the differences of the samples were reflected in the first and second principal components jointly. As shown in Figure 3, when TG enzyme supplemental levels were different, the distribution regions of fish sausage samples in the five groups did not overlap, indicating obvious flavor differences between groups. The samples with a TGase addition of 0.24 g were farther apart from the other groups, indicating that the flavor of the samples with a starch addition of 0.24 g was more prominent compared with the other groups. Combined with the gel strength and sensory evaluation results, the appropriate amount of starch addition was determined to be 0.24 g. The specific amount of starch addition was determined via response surface testing.

#### 3.2.4. Response Surface Testing to Optimize the Process Formulation of Low-Fat *Nemipterus virgatus* Fish Sausage

Based on the single-factor test, the response surface test factors and levels were set according to Table 8, and the mathematical model was developed using the Box–Behnken design, with fat substitute addition (A), TGase addition (B), and fat addition (C) as independent variables, and +1, 0, and −1 as high, medium, and low levels, respectively. The response value (R1) was based on the weighted average composite score, and the maximum gel strength of 858.62 g·cm and the highest overall sensory score of 79.273 for the low-fat fish sausage samples that were used as the reference. Comprehensive score = (N1/858.62) × 20% + (N2/79.273) × 80%. The results of the test are shown in Table 9. Using the software of design-expert 7.0, the regression equation was obtained as follows: R1 = 0.9717 + 0.0349 A − 0.0291 B + 0.0081 C + 0.0057 AB − 0.0078 AC − 0.0049 BC − 0.0427 A^2^ − 0.0983 B^2^ − 0.0734 C^2^.

#### 3.2.5. Establishment of the Regression Equation and Experimental Analysis of the Process Formulation of Low-Fat *Nemipterus virgatus* Fish Sausage

The results of ANOVA and significance analysis are shown in Table 10. The model *p* < 0.0001 indicates that the regression equation model is highly significant. The number of fat substitutes (A) and TGase (B) reached extremely significant levels, and the quadratic terms A_2_, B_2_, and C_2_ also reached extremely significant levels. The misfit term *p* = 0.4529 > 0.05, which is not significant, indicates that the model fits the test well and can predict the test results. The correlation coefficient R^2^ = 0.7979 and the corrected coefficient of determination RAdj^2^ = 0.9424 indicate that less than 2% of the variables cannot be explained via the model. The regression equation can better explain the true relationship between factors and response values. The signal-to-noise ratio of 14.6018 > 4 indicates that the model fit and credibility are good, and the model can be used to predict and optimize the test results.

#### 3.2.6. Response Surface Analysis and Optimization

**Figure 4:**
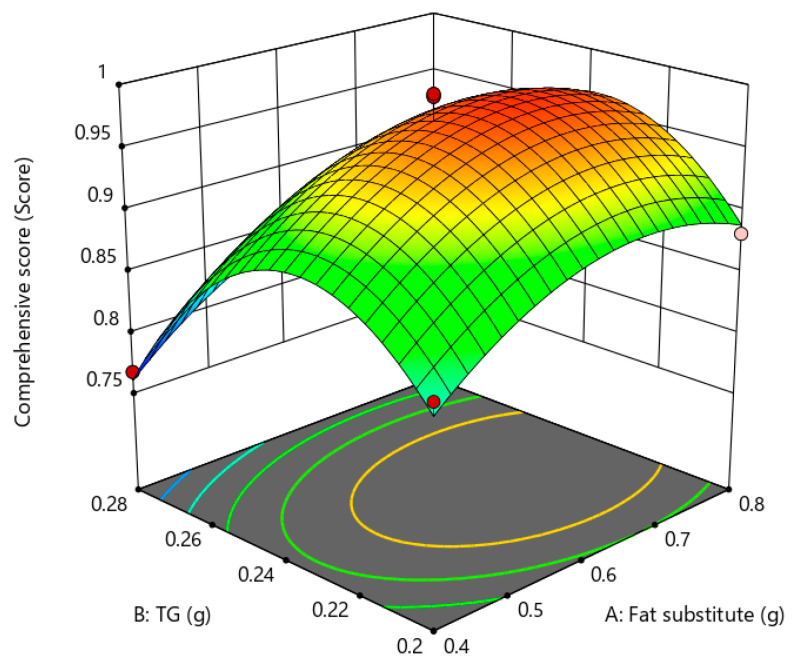
Effect of fat substitute and TGase on gel strength and sensory quality of low-fat fish sausage.

**Figure 5:**
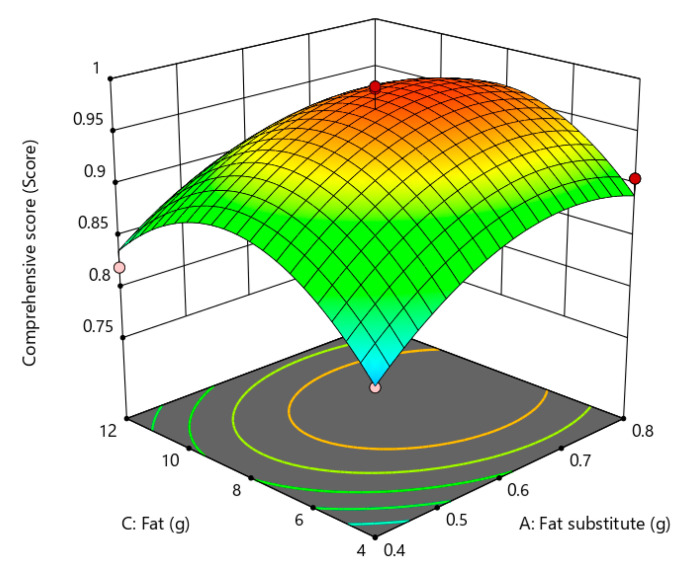
Effect of fat substitute and fat on gel strength and sensory quality of low-fat fish sausage.

**Figure 6:**
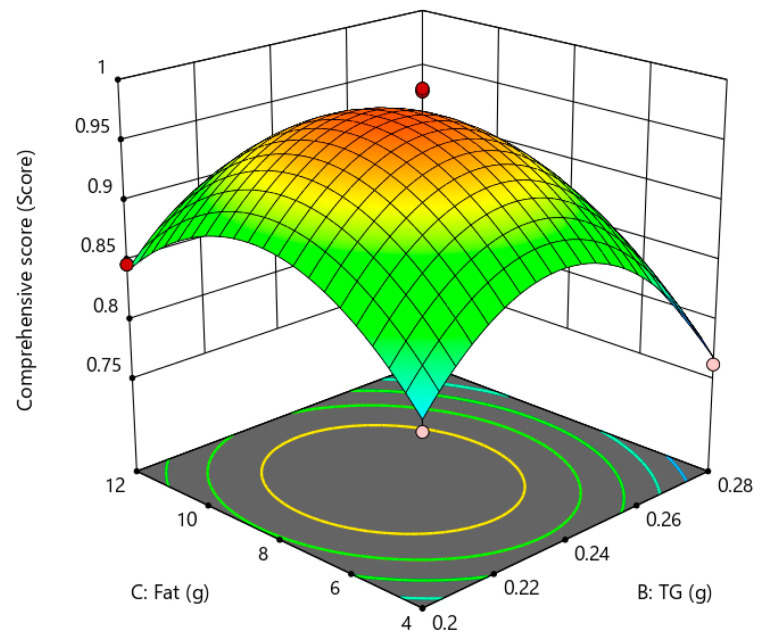
Effect of fat and TGase on gel strength and sensory quality of low-fat fish sausage.

Response surface is a three-dimensional spatial surface that presents the interaction relationship of each test factor drawn according to the regression equation, which can examine the magnitude of response values of variables and clarify the interrelationship of variables. According to the contour plot and response surface, the shapes and characteristics of the fitted response surfaces were observed, and the effects of the test factors A (amount of fat substitute), B (amount of TGase), and C (amount of fat) on the gel strength and sensory scores of low-fat fish sausage were analyzed as shown in Figure 4, Figure 5 and Figure 6. The steepness of the curve indicates a greater impact on the response value. From the magnitude of the *p* value in Table 11, it can be seen that the order of influence intensity of the selected factors on the response value is A (amount of fat substitutes) > B (amount of TGase) > C (amount of fat). Through the analysis, it is found that when the response values were set higher, the optimum values of A, B and C were 0.59 g, 0.245 g, and 8.03 g, respectively. Under this condition, the maximum value of the predicted comprehensive score was 0.9959.

#### 3.2.7. Validation of Optimal Conditions

To verify the reliability of response surface analysis, A (fat substitute), B (TGase), and C (fat) were set to 0.59 g, 0.245 g, and 8.03 g, respectively, under these conditions. The gel strength and sensory evaluation of low-fat fish sausage was measured and scored. The average value was obtained from three repetitions of the experiment. The average gel strength was 837.4 g·cm and the average sensory score was 79.424. The overall score was calculated as 0.9968, which was 0.18% different from the predicted value of the model, and the prediction condition was reliable. The gel strength and sensory score results of the low fat group and the control group are shown in Table 11.

### 3.3. Effect of Compound Fat Substitutes on the Quality of Nemipterus virgatus Fish Sausage

Table 11 shows that the addition amount of exogenous fat in the traditional *Nemipterus virgatus* fish sausages (control group) is 20 g (20%). Applying composite fat substitutes to the traditional *Nemipterus virgatus* fish sausages effectively replaces exogenous fat. The new low-fat *Nemipterus virgatus* fish sausages (low-fat group) obtained only 8.04 g (9.04%) of fat addition, and the addition amount of exogenous fat decreased by 54.8%.

At the same time, the new low-fat *Nemipterus virgatus* fish sausage made with the optimized formula has a smooth surface, a dense and uniform pore-free structure, good elasticity, a light white color, a meat aroma, and moderate bitterness and is juicy. The sensory score of the new low-fat fish sausage was significantly higher than that of the control group (*p* < 0.001), reaching 79.4, indicating that the application of composite fat substitutes did not reduce the flavor and sensory quality of traditional fish sausages but improved the sensory quality of *Nemipterus virgatus* fish sausages to a certain extent.

### 3.4. Discussion

In the single-factor experiment of optimizing the formula of a new low-fat fish sausage, this study mainly focused on sensory evaluation, supplemented with electronic nose instrument analysis, to analyze the effects of exogenous fat, compound fat substitute, and TGase on the sensory quality and flavor of fish sausage. In response to surface and orthogonal experiments, an electronic nose is not used to assist in analyzing the flavor of fish sausage. Although electronic noses have the advantages of being able to perceive very small odor differences, accurately analyze and judge subtle odor features, and typically do not require sample processing or destruction to maintain sample integrity, electronic noses have the disadvantage of not being able to determine the types and quantities of flavor substances qualitatively and quantitatively. The response surface and orthogonal experimental design resulted in many groups, and the PCA images obtained via the electronic nose analysis did not effectively distinguish the flavors of these groups. In addition, to obtain the flavor changes in the new low-fat fish sausage, it is necessary to further combine the electronic nose with the electronic tongue or use instruments such as GC-MS (Gas Chromatography Mass Spectrometer) [52,53,54], GC-IMS (Gas Chromatography Ion Mobility Spectrometer) [54,55], HS-SPME-GC-MS (Headspace Solid Phase Microextraction Gas Chromatography Mass Spectrometer) [56], etc., for qualitative and quantitative analysis.

At present, many scholars have conducted research on fat substitutes and successfully reduced the fat content in meat products by 1.46–75%. Navo first used cross-linked casein gel as a fat substitute to replace 50% of the fat in salami sausages [57]. Some studies have used soybean protein to make fat substitutes, successfully replacing 18–75% of the fat content in minced beef pie [14], sausage, and meat pie [58] while ensuring the application of sensory quality of the products. Zhou [59] replaced 60% of pork fat in chicken sausage with a compound fat substitute, 1% polysaccharide compound emulsion gel (2 g carrageenan, 1% kelp water extract polysaccharide, 0.3 g sodium caseinate), and the sensory score was not lower than that of the control group. Jiménez Colmenero [60] used the stable “oil in water” emulsion formed by sodium caseinate, soy protein isolate, and microbial transglutaminase emulsified olive oil to replace the pig fat in frankfurter sausage, and the fat content of sausage was reduced by 1.46%. Zhang [61] used compound fat substitutes (28.5% soybean oil, 30% whey protein, 0.75% carrageenan, and 40.75% water) as fat substitutes in sausage and found that there was no significant difference in texture properties between the substituted sausage and traditional high-fat products. In this study, the traditional surimi-based product *Nemipterus virgatus* fish sausage was selected as the research object, and a new low-fat fish sausage was first developed by replacing the exogenous fat with protein matrix fat substitutes. Compared with the traditional fish sausage, the content of exogenous fat in the product was reduced by 54.8%, and the sensory score was increased by 21.79%. The effect of different compounds replacing fat is related to its physical properties. Some can improve the color and structure of the product, while others can reduce the quality of the product. The proportion and types of compound fat substitutes are diverse, and the effects of substitute types and combination proportions on product texture, flavor, and acceptability need to be further studied.

## 4. Conclusions

In this study, enzyme-modified soybean isolate protein, marine fish collagen, and ovalbumin were selected to compound a protein matrix as a composite fat substitute and firstly applied to traditional surimi-based products to develop a new low-fat fish sausage. The best compound fat substitution (enzyme-modified soybean isolate:marine fish collagen:ovalbumin) ratio of 2:1:3 was obtained using the orthogonal test method with the sensory evaluation and gel strength value of the product as the index.

Using a combination of single-factor and response surface tests in combination with electronic nose technology, the optimal formulation of the novel low-fat *Nemipterus virgatus* fish sausage was determined as follows: 60.0 g of surimi, 0.59 g of complex fat substitute, 8.03 g of fat, 0.245 g of TGase, 3.5 g of sugar, 2.0 g of salt, 0.2 g of MSG, 0.3 g of I&G, and 10.0 g of water. The proportion of each component in the fish sausage is as follows: fish surimi, 67.52%; complex fat substitute, 0.66%; TGase, 0.28%; fat, 9.04%; starch, 6.75%; sugar, 3.94%; salt, 2.25%; monosodium glutamate, 0.23%; I&G, 0.34%; and water, 9%.

Compared with the original formulation, the content of exogenous fat in the new low-fat *Nemipterus virgatus* fish sausage was reduced by 54.8%. Meanwhile, the sensory score of fish sausage was increased by 21.79%, maintaining its good flavor and sensory quality. This study provides a theoretical basis for the development of low-fat surimi-based products and will contribute to the development of low-fat products. Low-fat surimi-based products, such as low-fat fish balls with meat in the center, low-fat fish cakes, and low-fat fish tofu, will be developed in the future.

## Figures and Tables

**Table 1:** The standard of sensory evaluation criteria of the *Nemipterus virgatus* fish sausage.

Test Items	Score Standard	Score/100
Taste (20)	Poor taste and rough texture	0~4
	Taste salty or light, no juiciness	4~8
	Medium salty and juicy	8~16
	Moderately salty, with a strong sense of juice	16~20
Smell (20)	Bad smell	0~4
	No meat flavor, too much fish smell	4~8
	Insufficient meat flavor, slightly fishy taste	8~16
	With mixed fragrance	16~20
Color and luster (20)	Off-white, lusterless	0~4
	Light white color, slightly yellowish	4~8
	Light white, slightly glossy	8~16
	Pale white, glossy	16~20
Tissue morphology (20)	Smooth surface, hollow, loose structure, with more free fat and water precipitation	0~4
	Smoother surface, with cavities, looser structure, slightly free from fat and water precipitation in the form of swimming tissue	4~8
	Smooth surface, small pores, denser and more uniform structure, no free fat or water precipitation	8~16
	Smooth surface, non-porous structure dense and uniform, no free fat or water precipitation	16~20
Elasticity (20)	No elasticity, chewing with gritty feeling and residue	0~4
	Poor elasticity, chewing with sandy feeling, no residue	4~8
	Elasticity general, chewing no sandy feeling no residue	8~16
	Good elasticity, chewing without gritty feeling, no residue	16~20

**Table 2:** Levels of factors for orthogonal experiment L_9_ (4^3^).

No.	Factor		
	A Collagen/g	B Soy Protein Peptide/g	C Ovalbumin/g
1	0.10	0.05	0.10
2	0.20	0.10	0.20
3	0.30	0.15	0.30

**Table 3:** Results of orthogonal experiment L_9_ (3^4^).

No.	Factor					Sensory Score/100	Comprehensive Score
	A Collagen/g	B Soy Protein Peptide/g	C Ovalbumin/g	D Empty Column	Gel Strength/g·cm		
1	0.10	0.05	0.10	1	152.790	60.32	0.6674
2	0.10	0.01	0.20	2	170.186	63.15	0.7024
3	0.10	0.15	0.30	3	191.755	65.26	0.7316
4	0.20	0.05	0.20	3	299.960	72.08	0.8398
5	0.20	0.01	0.30	1	555.559	78.08	0.9927
6	0.20	0.15	0.10	2	213.332	65.56	0.7424
7	0.30	0.05	0.30	2	444.133	79.50	0.9670
8	0.30	0.10	0.10	3	313.484	70.77	0.8313
9	0.30	0.15	0.20	1	410.687	77.43	0.9339
${\bar{K}}_{1}$	0.700	0.824	0.747	0865			
${\bar{K}}_{2}$	0.858	0.842	0.825	0.804			
${\bar{K}}_{3}$	0.911	0.803	0.897	0.801			
Range (R)	0.211	0.039	0.150	0.064			
Ordering	A > C > B						
Optimal combination	A_2_B_2_C_3_						

**Table 4:** Variance analysis of comprehensive score of fish sausage formula.

Factor	Mean Sum of Deviations (SS)	Freedom (df)	Variance (Ms)	F	Fɑ
A (Collagen)	7.2 × 10^−2^	2	3.6 × 10^−2^	2.000	F_0.05(2,2)_ = 5.14 F_0.01(2,2)_ = 10.9
B (Soy Protein Peptide)	2.0 × 10^−3^	2	1.0 × 10^−3^	0.056	
C (Ovalbumin)	3.4 × 10^−2^	2	1.7 × 10^−2^	0.944	
Error	0.11	6	0.055		
Total variation	0.218	12			

**Table 5:** Effects of fat substitute addition on gel strength and sensory quality of low-fat *Nemipterus virgatus* fish sausage.

No.	Fat Substitute Addition/g	Gel Strength/g·cm	Sensory Score/100	Comprehensive Score
0	0	353.324 ± 14.435	58.5 ± 1.6	0.7605 ± 0.0021 ^f^
1	0.4	403.028 ± 23.182	63.2 ± 3.2	0.8496 ± 0.0021 ^e^
2	0.6	498.587 ± 19.416	72.8 ± 2.4	0.9707 ± 0.0018 ^a^
3	0.8	553.075 ± 9.535	69.2 ± 1.7	0.9498 ± 0.0009 ^b^
4	1.0	576.541 ± 3.262	58.8 ± 2.8	0.8436 ± 0.0011 ^c^
5	1.2	584.095 ± 7.501	56.0 ± 4.1	0.8154 ± 0.0010 ^d^

Note: Different lowercase letters represent significant differences between different treatment groups (*p* < 0.05).

**Table 6:** Effects of fat addition on gel strength and sensory quality of low-fat *Nemipterus virgatus* fish sausage.

No.	Fat Addition/g	Gel Strength/g·cm	Sensory Score/100	Comprehensive Score
1	20.0	520.120 ± 6.539	62.7 ± 2.1	0.8734 ± 0.013 ^d^
2	16.0	518.526 ± 16.209	69.3 ± 2.9	0.9444 ± 0.015 ^c^
3	12.0	516.381 ± 8.067	70.5 ± 0.6	0.9569 ± 0.003 ^b^
4	8.0	518.876 ± 15.437	74.4 ± 1.5	0.9995 ± 0.009 ^a^
5	4.0	393.438 ± 6.406	65.2 ± 2.1	0.8522 ± 0.012 ^e^

Note: Different lowercase letters represent significant differences between different treatment groups (*p* < 0.05).

**Table 7:** Effects of TGase addition on gel strength and sensory quality of low-fat fish sausage.

No.	TGase Addition/g	Gel Strength/g·cm	Sensory Score/100	Comprehensive Score
0	0	400.672 ± 11.189	62.0 ± 1.3	0.7781 ± 0.013 ^e^
1	0.12	500.966 ± 28.933	60.7 ± 1.1	0.7872 ± 0.011 ^d^
2	0.16	624.958 ± 19.320	68.7 ± 0.9	0.9046 ± 0.008 ^c^
3	0.20	783.499 ± 10.500	70.0 ± 0.9	0.9561 ± 0.006 ^b^
4	0.24	853.221 ± 20.234	72.5 ± 1.2	1.000 ± 0.009 ^a^
5	0.28	663.221 ± 20.234	52.4 ± 0.9	0.7337 ± 0.012 ^f^

Note: Different lowercase letters represent significant differences between different treatment groups (*p* < 0.05).

**Table 8:** Factors and levels of response surface methodology.

Level.	Factor		
	A Fat Substitute Addition/g	B TGase Addition/g	C Fat Addition/g
−1	0.4	0.20	4.0
0	0.6	0.24	8.0
1	1.2	0.28	12.0

**Table 9:** Experimental design and results of Box-Behnke.

No.	Fat Substitute Addition/g	TGase Addition/g	Fat Addition/g	Gel Strength/g·cm	Sensory Score/100	Comprehensive Score
	A	B	C			
1	0.8	0.24	12	712.910	71.91	0.8918
2	0.6	0.24	8	803.620	76.89	0.9631
3	0.6	0.28	12	764.620	60.000	0.7836
4	0.6	0.24	8	854.047	78.667	0.9928
5	0.6	0.28	4	598.730	61.667	0.7618
6	0.8	0.2	8	653.150	72.333	0.8821
7	0.6	0.2	4	497.422	68.422	0.8064
8	0.6	0.24	8	838.278	79.273	0.9953
9	0.4	0.2	8	563.822	70.333	0.8411
10	0.6	0.24	8	858.620	78.333	0.9905
11	0.6	0.24	8	795.435	76.435	0.9566
12	0.4	0.28	8	667.961	60.667	0.7678
13	0.4	0.24	12	568.240	68.240	0.8210
14	0.8	0.28	8	776.475	64.475	0.8315
15	0.6	0.2	12	578.632	70.667	0.8479
16	0.4	0.24	4	497.160	68.160	0.8037
17	0.8	0.24	4	556.774	70.000	0.9064

**Table 10:** ANOVA for response surface regression model.

Source	Sum of Squares	df	Mean Square	F-Value	*p*-Value	Significant
Model	0.0959	9	0.0107	30.09	<0.0001	**
A	0.0097	1	0.0097	27.51	0.0012	**
B	0.0068	1	0.0068	19.14	0.0033	**
C	0.0005	1	0.0005	1.50	0.2608	
AB	0.0001	1	0.0001	0.3639	0.5654	
AC	0.0002	1	0.0002	0.6787	0.4372	
BC	0.0001	1	0.0001	0.2741	0.6168	
A²	0.0077	1	0.0077	21.72	0.0023	**
B²	0.0407	1	0.0407	114.96	<0.0001	**
C²	0.0227	1	0.0227	64.14	<0.0001	**
Residual	0.0025	7	0.0004			
Lack of Fit	0.0011	3	0.0004	1.08	0.4529	Not significant
Pure Error	0.0014	4	0.0003			
Cor Total	0.0983	16				
R^2^	0.7979					
Adjusted R^2^	0.9424					
Intercept	0.9717					
Adeq Precision	14.6018 > 4					

Notes: *p* < 0.001 indicates highly significant. 0.01 < *p* < 0.05 indicates significant. ** indicates highly significant.

**Table 11:** Sensory score and texture of low fat *Nemipterus virgatus* fish sausage.

Test Group	Fat/g	Gel Strength/g·cm	Sensory Score/100
Control group	20.0	276.714 ± 18.272 ^b^	62.1 ± 2.2 ^b^
Low-fat group	8.03	837.400 ± 12.89 ^a^	79.4 ± 1.7 ^a^

Note: Different lowercase letters represent significant differences between different treatment groups (*p* < 0.05).

## Data Availability

Considering the privacy of the respondents involved in the study, the data underlying this paper cannot be shared publicly. Datasets generated and/or analyzed during the current study will be shared with the corresponding authors upon reasonable request.
